# Supplementary material for: Content-rich biological network constructed by mining PubMed abstracts
Source: BMC Bioinformatics. 2004 Oct 8;5:147. doi: 10.1186/1471-2105-5-147 (PMC528731; doi:10.1186/1471-2105-5-147)
Supplement: Additional File 5 — The original Chilibot query results of the term "long-term potentiation (LTP)" and 22 other terms, limiting the latest references analyzed to the years 1990, 1995, 2000, and 2004. [file 1471-2105-5-147-S5.bz2 › chilibotAdditionalFile5/ltp2004/html/PI-3K_NMDA.html]

 


 **PI-3K** and **NMDA** 
  
Found 21 abstracts in PubMed,  **21 abstracts were retrieved and analyzed**.  


---

 Search Google  |
 PDF files only 
|  EDU domain only 

---

**Interactive relationship** (e.g. stimulation, inhibition, etc)

- Inhibition of apoptosis by  **NMDA**  was blocked by the phosphatidylinositol 3 kinase PI 3 kinase  [ **PI-3K** ]  inhibitor LY294002, but it was unaffected by the mitogen activated protein kinase kinase inhibitor PD 98059.  Ref: 9756898 J Biol Chem, 1998
- These data demonstrate that the neurotrophic action of  **NMDA**  and its inhibition by ethanol are mediated by alterations in the activity of a PI 3 kinase  [ **PI-3K** ]  dependent antiapoptotic signaling pathway.  Ref: 9756898 J Biol Chem, 1998
- PI3 kinase  [ **PI-3K** ]  blockade caused a loss of synapse specificity of LTD that was dependent on the co activation of  **NMDA**  type glutamate receptors NMDARs and metabotropic glutamate receptors mGluRs, and involved release of calcium from intracellular stores.  Ref: 12161757 Nat Neurosci, 2002
- Our results show that PI 3 kinase  [ **PI-3K** ]  Akt is activated by either  **NMDA**  or BDNF displaying differential kinetics.  Ref: 12373512 Amino Acids, 2002
- Therefore, the PI 3 kinase  [ **PI-3K** ]  Akt pathway is co activated by  **NMDA**  and TrkB receptors.  Ref: 12373512 Amino Acids, 2002
- ... the PI 3 kinase  [ **PI-3K** ]  Akt pathway is antiapoptotic in RGC death induced by  **NMDA** .  Ref: 12506100 Invest Ophthalmol Vis Sci, 2003
- The p38 MAP kinase and PI 3 kinase  [ **PI-3K** ]  Akt pathways are involved in signal transduction after excessive stimulation of  **NMDA**  receptors in the retina.  Ref: 12506100 Invest Ophthalmol Vis Sci, 2003
- We propose that  **NMDA**  receptor stimulation can activate Erk1 2 and Akt signalling pathways in a PI 3 kinase  [ **PI-3K** ]  dependent manner which may target CREB in the nucleus.  Ref: 11902114 J Neurochem, 2002
- Consistent with the notion that ERK activation by  **NMDA**  receptor stimulation is PI3 kinase  [ **PI-3K** ]  dependent, the PI3 kinase  [ **PI-3K** ]  inhibitor wortmannin partially inhibited ERK2 activation induced by bath application of  **NMDA**  and strongly suppressed ERK2 activation by high frequency synaptic stimulation.  Ref: 12736339 J Neurosci, 2003
- We now report that  **NMDA**  receptor activation of Erk1 2 was also blocked by inhibitors of PI 3 kinase  [ **PI-3K** ]  LY 294002, wortmannin.  Ref: 11902114 J Neurochem, 2002
- PI3 kinase  [ **PI-3K** ]  activity, which is stimulated by growth factors, is closely related to protective effects against NO and  **NMDA**  mediated toxicity in cortical neurons, especially those isolated from SHRSP.  Ref: 9166279 Lab Invest, 1997
- Inhibition of neuronal survival by ethanol corresponded with a marked reduction in the capacity of  **NMDA**  to increase the concentration of intracellular calcium, phosphorylate IRS 1, and activate PI 3 kinase  [ **PI-3K** ] .  Ref: 9756898 J Biol Chem, 1998
- The antiapoptotic action of  **NMDA**  was associated with an increase in the tyrosine phosphorylation of insulin receptor substrate 1 IRS 1, an increase in the binding of the regulatory subunit of PI 3 kinase  [ **PI-3K** ]  to IRS 1, and a stimulation of PI 3 kinase  [ **PI-3K** ]  activity.  Ref: 9756898 J Biol Chem, 1998

- :-)

  **Parallel relationship** (e.g. studied together, co-existance, homology, etc.)

  - **NMDA**  receptor antagonist, phosphatidylinositol 3 kinase PI 3 kinase  [ **PI-3K** ] , and MAP kinase inhibitors that blocked the acquisition also blocked the extinction of conditioned fear.  Ref: 12967993 J Neurosci, 2003
  - BDNF increased phosphorylation of Akt, a target of PI 3 kinase  [ **PI-3K** ] , and  **NMDA**  also induced Akt phosphorylation, but only after an exposure that was long enough to induce BDNF expression.  Ref: 10212287 J Neurosci, 1999
  - In the absence of extracellular calcium,  **NMDA**  was unable to prevent apoptosis or to phosphorylate IRS 1 and activate PI 3 kinase  [ **PI-3K** ] .  Ref: 9756898 J Biol Chem, 1998
  - PURPOSE To investigate the involvement of the p38 mitogen activated protein MAP kinase and phosphatidylinositol 3 PI 3 kinase  [ **PI-3K** ]  Akt signaling pathways after pathologic stimulation by N methyl D aspartate  **NMDA**  receptors of retinal neurons in vivo.  Ref: 12506100 Invest Ophthalmol Vis Sci, 2003
  - PI3 kinase  [ **PI-3K** ]  inhibitors also block activation of ERK by  **NMDA**  receptor stimulation, suggesting that PI3 kinase  [ **PI-3K** ]  inhibitors block LTP because PI3 kinase  [ **PI-3K** ]  is an essential upstream regulator of ERK activation.  Ref: 12736339 J Neurosci, 2003
  - Inhibitors of both phosphatidylinositol 3 kinase PI3 kinase  [ **PI-3K** ]  and MAPK ERK mitogen activate protein kinase extracellular signal related kinase activation inhibit  **NMDA**  receptor dependent long term potentiation LTP.  Ref: 12736339 J Neurosci, 2003
  - METHODS  **NMDA**  2 200 nmol, SB203580 0.2 10 nmol, an inhibitor of p38 MAP kinase, LY294002 6 nmol, an inhibitor of PI 3 kinase  [ **PI-3K** ] , or control solution was injected into the vitreous of Long Evans rats.  Ref: 12506100 Invest Ophthalmol Vis Sci, 2003
  - While one interaction is involved in the inhibition of  **NMDA**  receptor gated currents, the other is implicated in the attenuation of  **NMDA**  receptor mediated excitotoxicity through a PI 3 kinase  [ **PI-3K** ]  dependent pathway.  Ref: 12408866 Cell, 2002
  - The kinetics of BDNF and  **NMDA**  mediated activation of PI 3 kinase  [ **PI-3K** ]  Akt suggests that they have different roles in intraneuronal time related events.  Ref: 12373512 Amino Acids, 2002
  - **NMDA**  reversed H 2 O 2 mediated tyrosine phosphorylation of IRS 1 and its association with PI 3 kinase  [ **PI-3K** ] .  Ref: 11279262 J Neurochem, 2001
  - Neurons were subjected to several apoptotic paradigms, including serum deprivation, serum deprivation combined with exposure to  **NMDA**  receptor antagonists, or treatment with PI 3 kinase  [ **PI-3K** ]  inhibitors.  Ref: 10729337 J Neurosci, 2000
